# Address to the Graduates in Medicine at the University of Buffalo, April 27, 1853

**Published:** 1853-07

**Authors:** Frank H. Hamilton

**Affiliations:** Professor of Surgery


ART. II.—*Address to the Graduates in Medicine at the University of Buffalo, April* 27, 1853. By FRANK H. HAMILTON, M. D., Professor of Surgery.*

What do you suppose, gentlemen, to be the meaning and value of these diplomas which this university has now granted to you? Let it be certain that we understand them.

They attest that you, being, upon careful examination, found qualified, have been admitted to the degree of "Doctor in Medicine." They constitute, under the laws of this state, a license to practice medicine and surgery, investing you therefore with new rights and privileges. But for what purpose are these special rights and privileges conferred? and it is this which I am afraid you do not know.

Men are licensed to buy and sell merchandise; to build bridges; to construct turnpikes, canals, and railroads; to open theaters, circuses, race courses, saloons for gaming and drinking, and in all this you understand the object to be one. It is gain! To make money and get rich; honestly if they can, but at all events to get rich. They make no secret of their purpose. Each man has considered well the chances, and he has at length taken out that license by which, under approval of his conscience, he believes this object can be most certainly and speedily attained.

Have you sought a license to practice medicine and surgery from such motives? and do you understand that to this end we have granted you academic honors and the witness of our seal? solely as a means of getting rich?

Then do I feel myself instructed to disabuse you at once of your unfortunate mistake; and I must tell you plainly and without much waste of words, you have totally misapprehended our meaning, and the value of these diplomas. You have spent much time, and labor, and money, I fear, for nothing.

If you desire the gauds and trappings of wealth; if you sigh for the day when you shall possess lands and houses; if you long to look upon large chests full of precious gold which you may call all your own; nay more, if you would live at ease, and dying you would know that you had left to your family that competence which shall secure them from want—why then, turn

* It is proper to state that this address has been furnished for publication in this Journal at the Editor's solicitation. Having had the pleasure of listening to it while delivered by the author, we were desirous that our readers should have the same gratification, so far as this may be afforded by its perusal.

back! It may not be even now too late. Tear up those useless parchments, and with a brave heart begin again.

It will never do, my good fellows; you have entered the wrong door. Yonder is your way! To the right; to the left; to the field; to the counter; to the bar; to the forum; to the mines go. Go where you may lift the hod; go heave the hammer, and wield the sledge; go anywhere—but where we conduct you.

No, the diploma of the ale-house has but one meaning. It is an authority to sell for gain. To sell malt for money. And no man shall dare to interrogate the malt, nor inquire whether it carries into the thirsty veins of its consumers health or life; for good or for bad, it is a lawful trade, and a money making. *Caveat emptor.*

So also this your diploma has but one meaning. It is a command to give for mercy's sake; to give health and life; to lengthen the threads and mitigate the pains of this present existence. Without one word of condition expressed or implied that the world will return you wealth or even honor. Missionaries are you, ordained and sent abroad, to minister to your fellow-men. It never has been, and never can be, that any mere selfish, sordid or mercenary purpose should find a place in the heart of the true physician.

Such purposes and sentiments are as incompatible with a faithful performance of the duties now intrusted to you, as they would be with the obligations enjoined upon a missionary of the cross, or of any other minister of the Gospel. A mercenary physician and a mercenary clergyman, are alike unintelligible and parodoxical. They have alike mistaken their calling; or they have obtained their commissions surreptitiously, and hold them under a false pretense.

No doubt a physician has a right to be rich: nobody has, perhaps, a better right: and some physicians are in the receipt of annual incomes which secure to them ease and elegance. But the number of these in proportion to the whole, is exceedingly small—too small to warrant any man in regarding our profession as one of the roads to wealth. And whether this be so or not, whoever practices medicine and surgery for no other purpose than to make money—or with this as his chief purpose, is a positive curse to the people whom he professes to serve, and unworthy the honorable profession which he assumes to represent.

For my colleagues, therefore, I charge you not to be deceived, nor willfully to deceive us, while, speaking in their stead, I counsel you, not how to make money by your profession, nor, indeed, how to get practice, that is a matter which ought more to concern others than yourselves—but only how you can best serve those whom chance or choice has intrusted to your care.

If it were actually true that you are entitled to regard the practice of medicine in the light of a commercial adventure, and every consultation and prescription as an ordinary business transaction in which the first consideration ought to be whether it would prove remunerative in a pecuniary point of view, then I confess to you, frankly, I would not have you over-scrupulous in matters of taste, or of propriety, or of conscience even. I would counsel you in the language of Radcliffe to Mead, when the latter was about to commence practice: "There are two ways, my boy, for a physician to treat his patients, either to bully or cajole them. I have taken the former course, and have done pretty well as you see; you may take the latter and perhaps do equally as well."

The world does not lack for illustrations of the complete success of either of these modes; and I think it a matter of indifference which you choose to adopt.

In such a case I would charge you somewhat after this manner: Sirs, here are your licenses; there are your victims! In the trade which you are about to commence experience has proven that knavery is often most successful; by which I mean to say, that it pays best. You will, therefore, practice such deceptions and impositions as you shall judge expedient, without much fear of exposure, and with a tolerable certainty of a fair cash return. "Populus vult decipi, decipiatur"—or as it may read with a pretty free rendering—"All the world's an ass and he is a fool that does n't ride it." This you will find a very convenient maxim, and particularly comfortable for the rider. You will not fail to adopt, and apply it to practice whenever a suitable opportunity presents. And whenever one ass is tired, you will find another with his saddle, bridle, and blinders on. Ride him also.

If you would be advised as to books, read Don Quixote. Sydenham will tell you if you should read anything else. I think not. The less you know of medicine the better; and it is probable that all kinds of learning will prove a useless, and sometimes a troublesome incumbrance.

In short, if you would speculate advantageously upon the pains, and sufferings, and dying agonies of your fellow-men, copy the examples which, without much pains to look, you can see everywhere around you. Renounce sense as well as science, honor and honesty, and with a shameless impudence practice wholesale upon human credulity.

Finally, and I am sure you will not think me unreasonable, renounce, also, the title which this your alma mater has now conferred upon you. Adopt any new title or name which may suit your fancy or interest, but let a decent respect for the mother who has nourished you, and whom you cannot certainly wish to wrong, preserve her from the mortification of being compelled to recognize and acknowledge her recreant and disgusting offspring.

Then we have done with each other, and no obligations remain. You wished to get rich, and I have told you how it may be done, so make the most of it—away—there's a purse full—take it—and may the Lord have mercy on your souls!

But, my beloved pupils, it is not thus we separate; I know you too well, and I repudiate the thought that any of you will ever thus prostitute his talents and his honors. Loftier motives inspire you in this happy evening of a long and laborious day: and if you are impatient for the morning, it is that with its first gray dawnings you may hasten to your homes, and prepare with humble confidence to enter upon the benevolent work which is before you. You wait now only for our parting counsels, and we must not detain you.

That you will be virtuous, honest and upright citizens, I venture to assume. That you will be industrious in your habits, attentive and faithful to your patients, I hope I may assume also; that you will be kind, humane, gentlemanly and courteous in all your dealings and associations, I think my acquaintance with each of you personally will warrant me in believing. These are attributes and qualifications which to every physician must be regarded as indispensable.

One thing remains of which I have not spoken, but which I regard as equally indispensable. *You must continue to study.*

I have some fears, however, that you will hereafter overlook the importance of this precept, and, perhaps, neglect it altogether. I must detain you, therefore, while I seek to enforce, by a brief argument, its necessity.

It would not be strange if you felt hurt and almost offended at my seeming want of confidence in your present intentions, and in your future strength of purpose. The advice is quite superfluous, and might have been spared, you think. Books have been to you, for years, almost your sole companions. They have stood by you, and talked to you; they have instructed you patiently and intelligently from the simplest elements of anatomy, physiology, and chemistry, on through all the most subtle questions of pathology and practice; and you feel toward them very much as a child feels toward a kind parent, or as a pupil feels toward a watchful and intelligent teacher. You love and respect them, and you are certain that hereafter you shall always be glad to lean upon them for advice, and to turn to them for counsel when doubts or embarrassments arise.

I sincerely hope, gentlemen, that this honorable attachment to your trusty old friends may never cease; but I confess I have my fears as to how it may turn out. I have seen so many young men who were studious in college, lose gradually their enthusiasm, and at last, after a few years, shut up their books entirely, that my confidence in the good resolutions of my best pupils is very much diminished. You cannot now know how great will be the temptation to idleness; and how little by little you will be induced, perhaps, to relinquish study. The lawyer must consult his books daily, because his practice must conform to the statutes and decisions as found in them. The clergyman, also, must read, because the incessant draft upon his intellect for new and original conceptions would exhaust the deepest cistern unless there was some mode of supply. It is not so, however, with the physician. His practice is under a vail, and need not be known to any but himself: and its accuracy, therefore, cannot be submitted to any test of authorities. He is not surrounded by a thirsty congregation for whom he must ply his brain as with a force-pump unceasingly. If the physician were required to preach his doctrines as well as to practice them, then you would be in less danger, since the world would soon discover of the physician who did not study, that his well was often dry, or that it contained only muddy and stagnant water, unfit for use.

I may hint to you also, that although the physician is not compelled to disclose the condition of his own mind to his patients, yet they are none the less the sufferers if he neglects to purify and enrich it by turning into its reservoirs every valuable suggestion which may come within his reach.

There is also another influence which, it is not unreasonable to suppose, may in some measure determine your habits—popular opinion. A large portion of the world believes that very little of medicine or surgery can be learned from books. *Medicus nascitur, non fit,* say they.

Nor is it at all strange that men should so think when we consider how little they can know of the extent and depth of our science. The majority of even intelligent men must have only the most inadequate conceptions of the complexity of the human frame; and they must know still less of its mysterious sympathies, and of the variety and complication of its diseases. To them it seems, as no doubt a few years since it seemed to you, that with a moderate intellect, and with a moderate expenditure of time and labor, the whole of medicine ought to be easily and thoroughly mastered.

Many a kind-hearted lady, and as many a simple-hearted man, believe that they are the fortunate possessors of an invaluable treasure, in the form of a small, unpretending, much worn and rather dirty piece of paper, containing an infallible recipe for "sore eyes." It is a kind of sacred trust, a leaf from the sybils' book, of which themselves and their families are made the honored depository and guardians. Under no circumstances of favor or necessity can they be induced to part even temporarily with the original paper. Yet we have often seen, that these excellent people will not hesitate to copy it for all their friends; nor do I doubt that they would make in this matter no difference between friends and enemies, if only they had "sore eyes." Now I venture to say that neither these kind-hearted ladies or simple-hearted gentlemen have ever much informed themselves upon this subject, and that they will be incredulous when we assure them that there are not less than fifty distinct maladies which must be included under this single generic term; and that the diseases of the eye and its appendages as written down and carefully described in our books, exceed eight hundred. In many of which experience has shown that the plans of treatment must be antagonistical; and in nearly all of which more or less modifications are necessary.

It would not be difficult to illustrate this to the satisfaction of our friends who are not medical men, and who have paid us the compliment of listening to our remarks. No student receives from this college a license to practice physic and surgery until he thoroughly understands this branch, which is but a moiety of the whole science. You, therefore, gentlemen, at least appreciate the truthfulness of my statements.

Say to one quarter of the world that four years is a short time to obtain a respectable knowledge of medicine and surgery, and one quarter of the world will cry you mercy for an unmitigated dolt, and tell you that they have known not a few who were born doctors; to whom the science of doctoring came as naturally as nursing or teething, and that natural-born doctors are as real as natural-born fools. Thus, the seventh son of the seventh son, is a doctor of necessity, and in the very nature of things could be nothing else, as the chronicles of England do all along testify: and in our own country and in our own day, there has been, as every one knows, a family of blessed memory, who were, from their cradles, "natural bone-setters."

Cures have been wrought, also, by persons who do not seem to possess any of these secret rays of divinity; and yet they have neither book knowledge, nor, one would say, knowledge of any kind!

But enough of this—the fact is notorious that no mean proportion of the world, even of the so-called intelligent, employ and have confidence in the skill of such men. They seem to believe, if they do not actually say, that learning is to the practical physician an incumbrance, and that stupidity and medical skill are convertible terms.

What shall a poor man do then? If he looks at this argument well in the face it says—books will not earn bread, nor will they provide clothing or shelter. Upon that stage of life upon which the physician acts his part, the harlequin is the most successful player, and wins the loudest applause: "here comedy carries the day." Why then shall any man rise early and sit up late to acquire that which makes only such poor returns! May not the mere pressure of necessity compel one at length to abandon his unprofitable studies? I think one of the Grecian orators has answered your question, when on another occasion he said: "For myself, I can conceive of no necessity more urgent to free souls than the pressure of dishonor."

A self-approving conscience,—this is the manna from heaven whose presence if one feels within he can stand erect and walk courageously forward, even though his purse and his stomach are empty.

To any physician, of proper sensibilities, the loss of a life which has been intrusted to his care, is a circumstance always sufficiently painful; but to the physician who is conscious that the life has been sacrificed to his own inexcusable ignorance of the resources of his art, such an event must bring both pain and remorse unmingled with consolation. I do not think money can pay for the bitter reflections which must occupy the secret chambers of this man's heart.

But you will be surprised to learn that some medical men have both indirectly and directly given sanction to this popular error. They hold, or assume to hold, in great respect, a maxim of which they are themselves the authors, and which they are never weary of repeating. "The best scholars are not always the best physicians." It is the dangerous influence of such men which especially justifies my alarm lest you should be seduced from your studies. Not the open enemies without the walls have you cause to fear, so much as the traitors within, to whose treacherous whisperings you will be compelled to listen.

I do not pretend to dispute the correctness of their favorite dogma, but I understand it as usually applied, to have a much broader significance than a fair and literal construction would warrant. It means, that, *other things being equal*, "the best scholars are not always the best physicians," and in this interpretation it conveys a most false and pernicious sentiment.

Clinical observation is necessary as well as study to the accomplishment of the medical student, but if either one is to be retained to the exclusion of the other, study, I affirm without doubt, is by far the most important and should have the preference. I grant you, however, that the man who has learned all that he knows by observation merely, is always the most confident and self-trusting—he will make the boldest physician and surgeon of the two: but I deny that he is therefore the safest. His very assurance ought to be regarded often as evidence of his complete ignorance of the character of the malady with which he has to contend. If the scholar is occasionally timid, and in certain cases hesitates to assume the responsibility alone, it is because like a skillful pilot, amid the tempest and the night, he recognizes the coast, and he knows that to guide the vessel safely between the rocks and over the deceitful shoals, will require the most careful handling of the helm. Blind ignorance is never timid, but steers right on through the narrowest straits and over the most dangerous seas, however dark the night, and however threatening the storm.

There are not wanting examples of these bold, self-taught, practical men, who, axe in hand, have hewn their way into the profession, always traveling by the most direct route. With the same good axe they continue to push on, hewing right and left, and smiting with sinewy blows whoever and whatever stands in their path. No pusillanimous fears waste their strength, no embarrassing doubts hold back their sturdy arms, but every time the weapon falls its mission is felt, and recoiling nature answers to the stroke.

How are these practical men made? These men in doublets who are told to sit on the right, while the "well-read men," as they are called by way of delicate contempt, are told to sit on the left! Have they like Minerva burst forth from the head of Jupiter complete in wisdom and with all their brass upon them? or have they had a normal incubation, and have they passed regularly from infancy to adolescence and from adolescence to manhood and maturity? I think they will be found generally to have had a distinct and natural beginning. They were not born of books or begotten of colleges; but, as they will attest themselves, they are the pure offspring and upgrowth of personal experience; and this is still their only schoolmaster.

*Personal* experience, you will take care to notice—for when they deny the value and authority of books, they virtually ignore the experience of all but themselves.

Whatever then "experience" may mean to the educated physican, whose first bedside observations were made under the instruction of skillful teachers; or who at least first learned, and then observed the application of the rules which he had received,—to these men certainly "experience" means only unguided *experiment;* the result being always as much at the caprice of chance as the turning of a dice.

All first experience is experiment, and books were written and continue to be written to save our fellow-men and ourselves from the necessity of being twice subjected to these fatal risks.

This curious question, then—"How are these practical men made?" has here its final solution. They are made, and they live and thrive too sometimes, by experiment.

It is well if at eighty they discover in their crucibles some few scanty facts which, if they had looked another way, they might have known at thirty. It is quite as often, we fear, that they die wholly unconscious that the science which they faithfully intended to practice was all their lives a century or more ahead of them.

Baron Wenzel confessed that he destroyed a hat-full of eyes before he learned how to extract the lens. Go, gentlemen, before you attempt to operate upon your brother's eyes, and ascertain from the Baron himself, how at last he learned to cut safely and successfully: there is for you and all a hat-full of experience, which, if you are wise, you will hasten to appropriate.

I ought not to omit to mention as prominent among the causes which induce physicians to relinquish study, and which may hereafter also influence you, the irregular habits and fatigues incident to the practice of our profession. I mention this rather as a kind of apology for ourselves, than with a view to the presentation of any arguments by which you shall be able to meet it.

So far as it goes, the excuse is valid; and you must yourselves judge how much it shall be permitted to rob you of one of your most cherished privileges, and how much it shall lessen the claims of an urgent duty. I have little doubt, however, but that the excuse is plead oftener than the necessities will warrant; and that it is made frequently a convenient cloak for indolence, or for that want of system perhaps, without which one's work is never done, even where there is nothing to do; but each day is so cramped, and follows upon the other so close, that not a moment of unoccupied time can anywhere be found.

After a just appreciation of the utility of study, nothing, it seems to me, so much encourages its prosecution as an ample and well-chosen library.

Agreeable books are like boon companions: the oftener we see their faces, the more we become attached to them, until at length, and almost insensibly, such is the mere force of custom, their society and conversation becomes indispensable to our happiness. While, on the other hand, if we thrust out social companions and refuse to entertain, or to be entertained by them, we soon acquire an indifference if not an actual distaste for their society.

A good library in an office argues better for the occupant than a rich surgical armamentarium or a shelf of well selected and handsomely labeled medicines. Both instruments and medicines are useful, and may be necessary to successful practice, but they are, after all, only agents for good or for evil, according as they may be directed by intelligence or ignorance. It is much easier indeed, my friends, to find excellent medicines than skillful doctors. "I know very well," said Asmodeus, "there are such things as good remedies, but I cannot say whether there are any good physicians." A wrought iron nail with a gimlet are better instruments in the hands of an educated surgeon, than the levator and trephine in the hands of the uneducated; and catnip with cayenne are more efficient remedies when directed by science, than hellebore or aconitine when prescribed by empiricism. Indeed, gentlemen, if your offices lack libraries, I would choose that they should lack also instruments and medicines, and patients too.

In the choice of books you must be guided by your own judgment as to what is really most valuable; as a general rule, however, I advise you to select monographs. Whoever writes a monograph writes usually what he understands, and he writes, also, because he believes he has made some discoveries or improvements worthy of being published. The writer of complete treatises, on the contrary, is often a writer by trade; a literary speculator. "He lards his lean books with the fat of others' works," and offers you a cheap volume of ill-digested compilations or unfair abridgments. Or, if the book is, perchance, written by a sound and sensible man—a man of the highest authority in his profession, he is nevertheless not authority in all things alike—no man can be. His complete treatise may be in the main excellent, but unfortunately in the midst of the wholesome grains there lie scattered here and there much chaff, and some blasted and poisonous seeds which cannot easily be recognized or sifted out—so the whole volume has to be thrown away. It is a just subject of congratulation, therefore, that such men as Sir Astley, Brodie, Mott, &c., seldom compose any thing but simple treatises on special topics.

I shall be pardoned, I hope, for inviting your attention specially to American books.

The archives of American medical literature are not very full. In some departments we can claim rank with any nation; while in most departments there is a palpable deficiency, and in a few a mortifying barrenness. On the whole the number of good American medical works issued during the last half century, is very limited as compared with the number issued by the English, French or Germans during the same period.

We will not believe that American physicians and surgeons are any less competent to this species of literature than foreign surgeons. We know they are not. In this country may be found men practicing physic and surgery with heads as clear, with minds as well cultivated, with practical experience as large, and with a zeal and patience as indomitable as may be found in any other part of the world. We know them—many of them personally—and we know the stuff of which they are made. We know also, personally, and have seen the practice, and have read the books of some of those transatlantic authors whose works occupy our shelves to the exclusion of American names.

Gentlemen—we say it not in boasting, nor with a desire to disparage our neighbors, but only in reply to the ungenerous foreigner who taunts us with the barrenness of our literature. We have among us a multitude more competent to write books than many, who, although but little known at home, have acquired a cisatlantic reputation. These men have not written, and will not write, so long as there is no international copyright law for their protection, and which shall enable them to realize a respectable compensation for their labors: so long as French, German and English books can be issued by our publishers at the cost of paper and type, we, who must figure in our time with the "oil," cannot compete successfully with foreign rivals. If the manufacture of American medical books is insignificant, it is not because we have not the material nor the workmen, but because the government does not at all protect or encourage this branch of industry.

We confess that the catalogue is small, yet it is respectable, and we do not require you to prefer altogether American books, or in any case to hesitate between two volumes of unequal merit—but only where the merit is plainly equal, in a spirit of just patriotism to give the preference to the home production.

You must take, also, one or more of the best medical serials. How can you expect to keep wing-and-wing with the age, unless you read the journals? You have seen what a vast amount of rich stores have accumulated already. For centuries, in every part of the civilized world, men of the ripest intellect and scholarship, have been toiling; and year after year the treasures of knowledge relating to the laws of life, health and disease, have been poured in, until the great repositories seem nearly full. And while, during the progress of your studies and in the course of instructions which have just closed, you have observed, day after day, some new leaf unfolded, you have exclaimed at length "how vast the book,"—you have been seized with astonishment at what was already known—yet all along you cannot but have marked the many points where abruptly our knowledge has terminated. You had gone with us down into the depth of the mines, but always you will remember, we left the miners still at work in every shaft, with lantern and pick, digging incessantly deeper and deeper.

Do you think their labors will accomplish nothing, and that no more discoveries will hereafter be made?

Look—every day a new vein will be struck, and every day from out some one of these thousand shafts some new specimens of valuable ore will come up.

Let then the egotist grope and feel about in the dark well of his own personal experience, and console himself with the few grains of spelter which he mistakes for gold; while you, having impressed into your service many skillful assayers, gather yellow sands from every bucket, and fill your warehouses easily with the precious ingots.

Before you leave us there are a great many things more I would like to say; but in this day of bustle a man must speak out quickly and briefly what he has to say, and stand off the track. Among the many things that I have not had time to mention, it occurs to me just now to remind you to keep a daily record of your cases: a sort of daily bulletin, in which you shall note the victories and the disasters of each day; with the causes of success or of failure.

I shall detain you no longer. My colleagues have instructed me thus to speak with you and to give you our parting words of advice and admonition. It was a duty imposed upon one of us by a plain rule of propriety, and also by a law of tradition—as a venerable and universally observed custom in all medical colleges. The Father of Medicine himself used to call about him his pupils, and enjoin upon them all a faithful performance of the trust which was soon to be reposed in them. The good old man embraced in his tender and paternal heart the whole human family, and he would permit, therefore, none to go out from his instructions until they had confirmed their intentions with a solemn covenant, and sealed them with the sacrament of an oath.

I would that on this occasion, gentlemen, I could have invoked the presence of the Coan sage; and that in my stead you might have heard his voice saying "Filii!"—and that while you listened, and all of us here present bowed to the prophetic sounds which should follow, there might come again from out two thousand years those words of strong and fervent inspiration, "*ars longa, vita brevis est.*"

What noble sentiments! what manly incentives! what classic eloquence breathe forever in these words of Hippocrates:

"Art is long, life is fleeting."

It sounds to me like the voice of a trumpet before the battle, whose martial notes nerve the soldier and make him impatient of delay—or like the first booming of the enemy's guns, which bends the finger to the firelock, and makes the sword leap in its scabbard.

From where you now stand look out upon all that horizon before you. The earth is fair, and the grass lies green upon the hillsides. But do you not see over every mountain and valley, over every city and plain, over every cot and castle there hangs a cloud? It is the shadow of the wings of Azrael, and of his ten thousand inexorable ministers of death. They are busy there at their silent, ceaseless work; and soon you shall hear, like the breaking of the sea-waves along the shore, the distant wail of the mourners; the light which flickers faintly from out the broken windows of the cottage shall cease, and the glimmer of the stained oriels shall be quenched with purple and sackcloth.

There is sickness and pain and death every where across this beautiful earth. Wherever man flies, there these unrelenting foes follow. In all latitudes and climates, abroad and at home, the weary, panting fugitive still turns and struggles in vain to elude their pursuit, or to shake off their hateful companionship. To us—in their extremity of despair, they cry for succor. God grant that from the doors of this temple of science, now opening to let you forth, there may go out to these sufferers a new beam of hope.

Farewell, gentlemen. There is much to be done and no time to lose. "Ars longa, vita brevis est," let this be your talisman and device.

"A voice replied far up the height
*Excelsior.*"

Stop not, until you stand upon the mountains' head! And when you fall, fall with your armor on. To receive at last into his own bosom the arrow which his skill has turned aside from another,—this is the compliment and crown of a physician's hopes.

